# Estimating Baseline Survival Function in the Proportional Hazards Model Under Monotone Hazards

**DOI:** 10.1007/s42519-026-00563-6

**Published:** 2026-05-26

**Authors:** Yunhong Wu, Rick Chappell

**Affiliations:** 1 Department of Biostatistics and Medical Informatics, University of Wisconsin-Madison, Madison, 53726 WI USA; 2 Department of Statistics, University of Wisconsin-Madison, Madison, 53715 WI USA; 3 Wisconsin Alzheimer’s Disease Research Center, Madison, 53792 WI USA

**Keywords:** Censoring, Truncation, Cox model, Breslow estimator, Monotone hazards

## Abstract

In biomedical studies where patient risk is known to increase over time, estimating the baseline survival function under the Cox proportional hazards (PH) model becomes particularly challenging when data involve delayed entry (left truncation) and incomplete follow-up (right censoring). This paper proposes a Breslow-type estimator for left-truncated and right-censored data under a monotone hazard assumption. By incorporating the Cox regression coefficients into Tsai’s monotone maximum likelihood estimator (MLE), we develop a covariate-adjusted monotone MLE that generalizes both Tsai’s univariate approach and Lopuhaä’s monotone estimator, which is restricted to right-censored data. Theoretically, we establish strong consistency of the estimator and derive its asymptotic distribution at a fixed point. Empirically, simulations confirm that our approach remains numerically stable with sparse early-time data, yields nearly unbiased survival estimates even in small samples, and achieves consistent efficiency gains from baseline covariate adjustment. We illustrate the method with an application to the Channing House data.

## Introduction

The analysis of time-to-event data is often complicated by left truncation and right censoring, which stem from late study entry and loss to follow-up, respectively. Crucially, left truncation imposes a distributional constraint by systematically excluding subjects who fail prior to entry, whereas right censoring reflects incomplete observation of event times when failures occur after the end of the follow-up period. A primary objective in such settings is to estimate the survival function under truncation and/or censoring. However, left truncation introduces biased sampling, as the observed data are conditional on the event time exceeding the truncation time. To address the selection bias introduced by delayed entry, standard methods such as the [11] (KM) estimator and the [5] proportional hazards (PH) model can be adapted through risk-set modification to include only individuals who have entered the study by the check-up times. However, when the support of the truncation distribution is wide, the modified risk sets can be extremely small at early times, making the survival estimates highly sensitive to a few observed failures. Due to the discrete hazard structure, nonparametric maximum likelihood estimators (NPMLEs) such as the truncated KM estimator proposed by [23] and the [18]- [1] estimator modified for left truncation can significantly underestimate survival probabilities. If all individuals at risk fail at a non-terminal time point, the NPMLEs may drop to zero prematurely – despite the presence of observed data beyond that point. Assuming a monotone hazard is an effective way to mitigate such issues, as the resulting monotone maximum likelihood estimator (MLE) is well defined even when the standard NPMLE fails to exist [7]. In practice, survival functions often exhibit increasing failure rates due to aging or disease progression, and events such as death, infection, or disease onset are typically associated with non-decreasing baseline hazards [15]. Motivated by these empirical patterns, [22] proposed a monotone MLE for left-truncated and right-censored data under the assumption of an increasing hazard rate. However, Tsai’s estimator does not incorporate covariates and therefore cannot accommodate multivariable analysis. Building on the Cox PH model, Lopuhaä and Nane proposed a monotone-baseline-hazard analogue of the [3] estimator that allows covariate adjustment, although their method applies only to right-censored data. In this paper, we present a more general estimator of the survival function that extends the approach of Lopuhaä and Nane to accommodate both left-truncation and right-censoring under monotone hazards, where the continuous baseline hazard function is assumed to be nondecreasing. By maximizing the partial likelihood and then projecting onto the space of monotone functions, the proposed estimator adopts an isotonic regression approach similar to that of Lopuhaä and Nane, but modifies the likelihood as well as the cumulative process to account for the conditional nature of the left-truncated samples. Without left truncation, our proposed estimator coincides with that of Lopuhaä and Nane. Alternatively, it can be interpreted as a covariate-weighted extension of the monotone MLE proposed by Tsai, reducing to Tsai’s estimator in the univariate setting. The simulation study demonstrates robust performance of the proposed method relative to existing methods in terms of bias, empirical standard error (ESE), and mean squared error (MSE) of the estimated survival probability at the true median survival time.

The remainder of this paper is organized as follows. Section 2 introduces the data structure and reviews standard methods, including the truncated KM estimator, the Cox model, and the Breslow estimator, followed by the formulation of our proposed method. Section 3 presents theoretical results on strong consistency and the pointwise asymptotic distribution of the proposed estimator, with detailed proofs deferred to the Appendices. Section 4 describes the simulation study conducted to evaluate the performance of our method in comparison with existing estimators. Section 5 applies these methods to the Channing House data, which records mortality among elderly residents in a retirement center [9]. Section 6 concludes with a discussion.

## Methods

### Data Structure

Throughout the paper, uppercase letters denote random variables, while lowercase letters indicate their corresponding realizations. Let *X* represent the event time (e.g., age at death) following the distribution *F* with an associated survival function S=1-F. Let f(x)=dF(x)/dx and λ(x)=f(x)/S(x) denote the probability density function and hazard function of *X*, respectively. Let (*T*, *C*) be the random variables for the left truncation time and right censoring time. We assume that (*T*, *C*) is conditionally independent of *X* given $\boldsymbol{Z}=(Z_1,...,Z_p)^T$, a *p*-dimensional vector of covariates. The joint distribution of (*T*, *C*) is denoted by *G*, with Pr(T<C)=1. The observed follow-up time is defined as Y=min(X,C), with corresponding censoring indicator $\Delta =\textbf{1}(X\le C)$, where $\textbf{1}{(\cdot )}$ is the indicator function. The conditional distribution and density functions of the observed time *Y* given $\boldsymbol{Z=z}$ are denoted by $F(y \mid \boldsymbol{z})$ and $f(y \mid \boldsymbol{z})$, respectively. The corresponding cumulative hazard function is defined as $\Lambda (y \mid \boldsymbol{z}) = -\log \left\{ 1 - F(y \mid \boldsymbol{z})\right\}$. Suppose the observed data are *n* independent and identically distributed vectors $\{(t_i,y_i, \delta _i, \boldsymbol{z}_i)\}_{i=1}^n$, each having the same joint distribution as $(T,Y,\Delta , \boldsymbol{Z})$ conditional on T≤X. Let $v_1<...<v_k$ denote the distinct ordered values from the set $\{y_1,...,y_n,t_1,...,t_n\}$, and let $y_{(1)}<...<y_{(N)}$ denote the distinct uncensored failure times, where $N=\sum _{i=1}^n\delta _i$.

### Truncated KM Estimator

The [11] estimator, also known as the product-limit estimator, is a cornerstone of nonparametric survival analysis. For event times subject to left truncation in addition to the usual right censoring, [23] proposed the truncated product-limit estimator of the survival function *S* as

1 $$\begin{aligned} \hat{S}(y) =\prod _{j: y_j \le y} \left( 1 - \hat{\lambda }^d(y_j) \right) = \prod _{j: y_j \le y} \left( 1 - \frac{d(y_j)}{n(y_j)} \right) , \end{aligned}$$

where

2 $$\begin{aligned} d(y_j)=\sum \limits _{i=1}^n \textbf{1}(y_i=y_j,\delta _i=1), \quad n(y_j)=\sum \limits _{i=1}^n \textbf{1}\{t_i\le y_j\le y_i\}, \end{aligned}$$

and $\hat{\lambda }^d(y_j)$ denotes the discrete hazard at $y_j$, defined as the estimated probability of failure at $y_j$ among individuals at risk. By modifying the risk set at each failure time to exclude individuals who have not yet entered the study, the above estimator $\hat{S}(y)$ serves as an analogue of KM estimator and reduces to the standard KM estimator for right-censored data whose $T_1=...=T_n=0$. Under standard assumptions such as independent truncation and censoring times, the truncated KM estimator $\hat{S}(y)$ is consistent.

### Cox Model

The [5] PH model extends the KM estimator by incorporating covariate information into the analysis of survival data. Assuming that covariates act multiplicatively on a nonparametric baseline hazard function, the Cox PH model specifies the conditional hazard function as follows

3 $$\begin{aligned} \lambda (y|\boldsymbol{z})=\lambda _0(y)\exp {(\boldsymbol{\beta }^T\boldsymbol{z})}, \end{aligned}$$

where $\boldsymbol{\beta }=(\beta _1,...,\beta _p)^T$ is a p-dimensional vector of regression coefficients, and $\lambda _0(\cdot )$ is an unspecified baseline hazard function. In the presence of censoring, inference is based on the partial likelihood on failed subjects among those at risk. Taking the logarithm of the partial likelihood yields

4 $$\begin{aligned} \ell (\boldsymbol{\beta })=\sum \limits _{i=1}^N \left[ \boldsymbol{\beta }^{T} \boldsymbol{z_i} -\log \left\{ \sum \limits _{j\in {R}_i}\exp (\boldsymbol{\beta ^T z_j})\right\} \right] , \end{aligned}$$

where $R_i=\{j:y_j\ge y_{(i)}\}$ represents the set of indices for individuals at risk at each observed failure time $y_{(i)}$. The maximum partial likelihood estimator $\boldsymbol{\hat{\beta }}$ is obtained by setting the first partial derivatives of the log-partial likelihood equal to zero, leading to the score equations:

5 $$\begin{aligned} \frac{\partial \ell (\boldsymbol{\beta })}{\partial {\beta _p}}=\sum \limits _{i=1}^N z_{ip}-\frac{\sum \limits _{j\in {R}_i} z_{jp}\exp (\boldsymbol{\beta }^T \boldsymbol{z}_j)}{\sum \limits _{j\in {R}_i} \exp (\boldsymbol{\beta }^T \boldsymbol{z}_j)}=0, \end{aligned}$$

which can be solved using the Newton-Raphson algorithm and the matrix of second partial derivatives. Similar to the truncated KM estimator, the Cox model can also accommodate left truncation by modifying the risk set to account for delayed entry, with $R(y)=\{j:t_j\le y\le y_j\}$ [13]. For later theoretical analysis, it is convenient to express the Cox partial likelihood using counting–process notation. Define the at–risk and counting processes

6 $$\begin{aligned} R_i(t)=\textbf{1}\{T_i\le t\le Y_i\}, \qquad N_i(t)=\textbf{1}\{Y_i\le t,\ \Delta _i=1\}. \end{aligned}$$

Then the partial log–likelihood with delayed entry can be written as

7 $$\begin{aligned} \ell _n(\boldsymbol{\beta })=\sum _{i=1}^n \int \left\{ \boldsymbol{\beta }^T \boldsymbol{Z}_i-\log \big (n S_n^{(0)}(\boldsymbol{\beta },t)\big )\right\} \, dN_i(t), \end{aligned}$$

where

8 $$\begin{aligned} S_n^{(k)}(\boldsymbol{\beta },t)=\frac{1}{n}\sum _{j=1}^n R_j(t)\exp (\boldsymbol{\beta }^T \boldsymbol{Z}_j)\, \boldsymbol{Z}_j^{\otimes k}, \qquad k=0,1,2. \end{aligned}$$

### Breslow Estimator

In the discussion of Cox’s paper, [3] suggested the simultaneous estimation of $\boldsymbol{\beta }$ and $\lambda _0(\cdot )$ by maximizing the joint likelihood function:

9 $$\begin{aligned} L(\boldsymbol{\beta },\lambda _0(\cdot ))=\prod \limits _{i=1}^n \left\{ \exp (\boldsymbol{\beta }^T \boldsymbol{z}_i)\lambda _0(y_i)\right\} ^{\delta _i}\exp \left\{ -\int _{0}^{y_i}\exp (\boldsymbol{\beta }^T \boldsymbol{z}_i)\lambda _0(t)dt\right\} . \end{aligned}$$

Treating $\lambda _0(\cdot )$ as piecewise constant between distinct uncensored failure times, i.e., $\lambda _0(y) = \lambda _i$ for $y_{(i-1)} < y \le y_{(i)}, i=1,\ldots ,N,$ where $y_0=0$, and following the convention of [10] in which each censored observation is considered censored at its preceding observed failure time, Breslow shows that $L(\boldsymbol{\beta }, \lambda _0(\cdot ))$ is maximized simultaneously at $\hat{\boldsymbol{\beta }}$ and the discrete hazard estimate

10 $$\begin{aligned} \hat{\lambda }_i=\frac{d(y_{(i)})}{(y_{(i)}-y_{(i-1)}) \sum \limits _{j\in R(y_{(i)})}\exp (\boldsymbol{\hat{\beta }}^T \boldsymbol{z}_j)}. \end{aligned}$$

By applying the profile likelihood approach through the extension of the [18]- [1] estimator, Breslow provides the NPMLE for the baseline cumulative hazard function $\Lambda _0(y)=\int _0^y\lambda _0(u)du$. The Breslow estimator, evaluated at the observed failure time $y_{(i)}$, is given by

11 $$\begin{aligned} \hat{\Lambda }_0(y_{(i)})=\sum _{k=1}^i\frac{d(y_{(k)})}{\sum \limits _{j\in R(y_{(k)})}\exp (\boldsymbol{\hat{\beta }}^T \boldsymbol{z}_j)}. \end{aligned}$$

Once both $\boldsymbol{\beta }$ and $\Lambda _0(\cdot )$ have been estimated, the survival function for a given covariate vector $\boldsymbol{z}$ can be predicted by

12 $$\begin{aligned} \hat{S}(y|\boldsymbol{z};\hat{\boldsymbol{\beta }},\hat{\Lambda }_0(\cdot ))=\exp \left\{ -\int _{0}^{y}\exp ({\hat{\boldsymbol{\beta }}^T \boldsymbol{z}})\hat{\lambda }_0(u) du\right\} =\exp \left\{ -\hat{\Lambda }_0(y)\exp ({\hat{\boldsymbol{\beta }}^T \boldsymbol{z}})\right\} . \end{aligned}$$

### A Semiparametric Monotone Maximum Conditional Likelihood Estimator

In the presence of left truncation and right censoring, inclusion of a subject requires survival beyond the entry time $t_i$. Accordingly, the likelihood contribution for subject *i* is conditioned on $X_i>t_i$, which is equivalent to dividing the unconditional likelihood contribution by the survival function evaluated at $t_i$ under the independent truncation assumption. The conditional likelihood of the observed data points $\{(y_i,t_i,\delta _i),i=1,...,n\}$ is

13 $$\begin{aligned} L_C\propto \prod \limits _{i=1}^n\{f(y_i)^{\delta _i}S(y_i)^{1-\delta _i}/S(t_i)\}=\prod \limits _{i=1}^n\{\lambda (y_i)^{\delta _i}S(y_i)/S(t_i)\}, \end{aligned}$$

where ∝ indicates extra terms involving the joint distribution G resulting from left truncation and right censoring. Plugging in (3) and (12), we can then re-write (13) as

14 $$\begin{aligned} \begin{aligned} L(\lambda _0(\cdot );\hat{\boldsymbol{\beta }})&\propto \prod \limits _{i=1}^n\{\lambda (y_i|\boldsymbol{z}_i)^{\delta _i}S(y_i|\boldsymbol{z}_i;\hat{\boldsymbol{\beta }},\lambda _0(\cdot ))/S(t_i|\boldsymbol{z}_i;\hat{\boldsymbol{\beta }},\lambda _0(\cdot ))\}\\  &=\prod \limits _{i=1}^n \left\{ \exp ({\hat{\boldsymbol{\beta }}^T \boldsymbol{z}_i})\lambda _0(y_i)\right\} ^{\delta _i} \frac{\exp \left\{ -\int _{0}^{y_i}\exp ({\hat{\boldsymbol{\beta }}^T \boldsymbol{z}_i})\lambda _0(u)du\right\} }{\exp \left\{ -\int _{0}^{t_i}\exp ({\hat{\boldsymbol{\beta }}^T \boldsymbol{z}_i})\lambda _0(u)du\right\} }. \end{aligned} \end{aligned}$$

This represents the conditional likelihood for $\lambda _0(\cdot )$, given the regression coefficients $\hat{\boldsymbol{\beta }}$ estimated from the Cox PH model with left-truncated and right-censored data. The conditional log-likelihood of (14) is thus

15 $$\begin{aligned} \ell (\lambda _0(\cdot );\hat{\boldsymbol{\beta }})=\sum \limits _{i=1}^n\delta _i \log \{\exp ({\hat{\boldsymbol{\beta }}^T \boldsymbol{z}_i})\lambda _0(y_i)\}-\sum \limits _{i=1}^n\int _{t_i}^{y_i}\exp ({\hat{\boldsymbol{\beta }}^T \boldsymbol{z}_i})\lambda _0(u)du. \end{aligned}$$

While the standard Cox model estimates $\boldsymbol{\beta }$ via the partial likelihood without specifying the baseline hazard function, Cox explicitly recognized-at J.W. Tukey’s suggestion-that monotonicity constraints could be applied to the baseline hazard.

Suppose that $\lambda _0(\cdot )$ is non-decreasing, it follows [16] that

16 $$\begin{aligned} \begin{aligned} \ell (\lambda _0(\cdot );\hat{\boldsymbol{\beta }})&\le \sum \limits _{i=1}^n \delta _i \log \{\exp ({\hat{\boldsymbol{\beta }}^T \boldsymbol{z}_i})\lambda _0(y_i)\}-\sum \limits _{i=1}^n \{(v_{l_i+1}-v_{l_i})\exp ({\hat{\boldsymbol{\beta }}^T \boldsymbol{z}_i})\lambda _0(v_{l_i}) \\&+ (v_{l_i+2}-v_{l_i+1})\exp ({\hat{\boldsymbol{\beta }}^T \boldsymbol{z}_i})\lambda _0(v_{l_i+1})+...\\&+(v_{\mu _i}-v_{\mu _i-1})\exp ({\hat{\boldsymbol{\beta }}^T \boldsymbol{z}_i})\lambda _0(v_{\mu _i-1})\}\\&=\sum \limits _{i=1}^n \delta _i \hat{\boldsymbol{\beta }}^T \boldsymbol{z}_i + \sum \limits _{i=1}^n \delta _i \log (\lambda _0(y_i))\\&-\sum \limits _{i=1}^{k-1}\sum \limits _{l\in R(v_i+)}\exp (\boldsymbol{\hat{\beta }}^T \boldsymbol{z}_l)(v_{i+1}-v_i)\lambda _0(v_i)\\&=\ell ^*(\lambda _0(\cdot );\hat{\boldsymbol{\beta }}), \end{aligned} \end{aligned}$$

where $v_{l_i}=t_i,v_{\mu _i}=y_i$, and $R(y+)=\{l:t_l\le y< y_l\}$. The problem of maximizing $\ell (\lambda _0(\cdot );\hat{\boldsymbol{\beta }})$ is equivalent to that of maximizing $\ell ^*(\lambda _0(\cdot );\hat{\boldsymbol{\beta }})$. Fixing $\hat{\boldsymbol{\beta }}\in \mathbb {R}^p$, the log-likelihood further simplifies to

17 $$\begin{aligned} \begin{aligned} \ell ^*(\lambda _0(\cdot );\hat{\boldsymbol{\beta }})&=\sum \limits _{i=1}^n \delta _i \log (\lambda _0(y_i))-\sum \limits _{i=1}^{k-1}\sum \limits _{l\in R(v_i+)}\exp (\boldsymbol{\hat{\beta }}^T \boldsymbol{z}_l)(v_{i+1}-v_i)\lambda _0(v_i)\\&=\sum \limits _{i=1}^{k-1}\left\{ s_i\log (\lambda _0(v_i))-\lambda _0(v_i)\right\} w_i, \end{aligned} \end{aligned}$$

where

18 $$\begin{aligned} w_i=(v_{i+1}-v_i)\sum \limits _{l=1}^n\exp (\boldsymbol{\hat{\beta }}^T \boldsymbol{z}_l)\textbf{1}\{t_l\le v_i< y_l\}, \qquad s_i=\frac{d( v_i)}{w_i}. \end{aligned}$$

Following the argument of Breslow, Marshall and Proschan, [4, 19, 22] and [15], the baseline hazard estimator $\hat{\lambda }_0$ maximizing (17) subject to $\lambda _0(v_1)\le ...\le \lambda _0(v_k)$ is defined by

$$\begin{aligned} \hat{\lambda }_0(y;\hat{\boldsymbol{\beta }})={\left\{ \begin{array}{ll} 0 \quad (y<v_1),\\ \hat{\lambda }_j \quad (v_j\le y < v_{j+1}; j=1,...,k-1),\\ \hat{\lambda }_{k} \quad (v_k\le y), \end{array}\right. } \end{aligned}$$

where

19 $$\begin{aligned} \hat{\lambda }_j=\max \limits _{1\le r \le j}\min \limits _{j\le s\le k-1}\frac{\sum \limits _{i=r}^s d(v_i)}{\sum \limits _{i=r}^s \sum \limits _{l\in R(v_i+)}\exp (\boldsymbol{\hat{\beta }}^T \boldsymbol{z}_l) (v_{i+1}-v_i)}. \end{aligned}$$

Similar to the KM estimator, $\hat{\lambda }_{k}$ is undefined when the largest failure time is censored. For simplicity, we follow the approach of Tsai and define $\hat{\lambda }_0(y;\hat{\boldsymbol{\beta }}) = \hat{\lambda }_0(v_{k-1};\hat{\boldsymbol{\beta }})$ for $y \ge v_k$. The above max–min formula follows from Theorem 1.10 in Brunk et al. and Theorem 1.4.4 in [21], where the value $\hat{\lambda }_j$ corresponds to the left derivative at $P_i$ of the greatest convex minorant (GCM) of the cumulative sum diagram (CSD), which consists of the points

20 $$\begin{aligned} P_i = \left( \frac{1}{n} \sum _{j=1}^{i} w_j, \; \frac{1}{n} \sum _{j=1}^{i} w_j s_j \right) , \quad i = 1, 2, ..., k-1, \end{aligned}$$

and $P_0 = (0, 0)$. For the x-coordinate of the CSD, it follows that

21 $$\begin{aligned} \begin{aligned} \frac{1}{n} \sum _{j=1}^{i} w_j&= \sum _{j=1}^{i} (v_{j+1} - v_j) \frac{1}{n} \sum _{l=1}^{n} \textbf{1}\{t_l\le v_j< y_l\} \exp (\boldsymbol{\hat{\beta }}^T \boldsymbol{z}_l)\\&=\int _{v_1}^{v_{i+1}} \int \textbf{1}\{t\le s\le u \} \exp (\boldsymbol{\hat{\beta }}^T \boldsymbol{z}) \, d\mathbb {P}_n(t, u, \delta , \boldsymbol{z}) \, ds \\&= W_n(\boldsymbol{\hat{\beta }}, v_{i+1}) - W_n(\boldsymbol{\hat{\beta }}, v_1), \end{aligned} \end{aligned}$$

where $\mathbb {P}_n$ denotes the empirical measure of the $(T_i, Y_i,\Delta _i,\boldsymbol{Z}_i)$. For the y-coordinate of the CSD, it can be seen that

22 $$\begin{aligned} \frac{1}{n} \sum _{j=1}^{i} w_j s_j =\frac{1}{n}\sum \limits _{j=1}^i d(v_j) = V_n(v_{i+1}). \end{aligned}$$

When $\hat{\boldsymbol{\beta }}=\boldsymbol{0}$ or all subjects share the same covariate vector (a single subgroup), our proposed estimator reduces to the Tsai estimator. This parallels how the Breslow estimator simplifies to the form of the KM estimate considered by Nelson [18]. The corresponding maximum conditional likelihood estimator of the covariate-specific survival function is given by $\hat{S}(y|\boldsymbol{z};\hat{\boldsymbol{\beta }},\hat{\Lambda }_0(\cdot ))$, as defined in (12), where the cumulative baseline hazard function can be estimated by

23 $$\begin{aligned} \hat{\Lambda }_0(y)=\int _{0}^y \hat{\lambda }_0(u) du = \sum \limits _{i=1}^{j-1} (v_i-v_{i-1})\hat{\lambda }_{i-1} + (y-v_{j-1})\hat{\lambda }_{j-1}, \qquad j=1,\ldots ,k, \end{aligned}$$

where $$v_0=0$$, $$\hat{\lambda }_0=0$$, and *j* is such that $$v_{j-1}\le y< v_{j}$$, with the estimates $$\hat{\lambda }_j$$ obtained from (19). By restricting the baseline hazard to a non-decreasing step function that is constant on each interval $$[v_{j-1},v_{j})$$ with $$\hat{\lambda }_{j-1}\le \hat{\lambda }_j$$, the underlying survival distribution is essentially a specialized form of a piecewise exponential distribution, leading to a continuous (though its derivatives may be discontinuous) version of the survival estimates.

## Theoretical Results

In this section, we establish the strong consistency and pointwise asymptotic distribution of the proposed estimator, with detailed proofs deferred to Appendices A and B, respectively. Throughout the proofs, let $\hat{\lambda }_n$ denote the proposed baseline hazard estimator (19) based on a sample of size *n*. Let $\boldsymbol{\beta }_0$ denote the true value of the regression parameter in the [5] PH model. We first introduce the following assumptions.

### Assumption 1

Let $G_1(t|\boldsymbol{z}) = \Pr (T \le t | \boldsymbol{Z}=\boldsymbol{z})$ denote the conditional distribution function of the truncation time *T*, and let $G_2(t|\boldsymbol{z}) = \Pr (C \le t | \boldsymbol{Z}=\boldsymbol{z})$ denote the conditional distribution function of the censoring time *C*. Define $a_F = \sup \{t: F(t|\boldsymbol{z})=0\}$ and $b_F = \inf \{t: F(t|\boldsymbol{z})=1\}$ as the endpoints of the support for the failure time distribution *F*. We assume *F* represents an increasing failure rate and is thus absolutely continuous. Under these definitions, it holds that $a_{G_1(\cdot |\boldsymbol{z})}< a_F< b_F < b_{G_2(\cdot |\boldsymbol{z})}$ for all $\boldsymbol{z}$.

### Assumption 2

For each $$y_0\in [a_F, b_F]$$, $$\text {pr}\,(T<y_0<C)>0$$.

### Assumption 3

There exists η>0 such that

$$ \mathbb {E}\!\left[ \sup _{\Vert \boldsymbol{\beta }-\boldsymbol{\beta }_0\Vert \le \eta } \Big \{(1+\Vert \boldsymbol{Z}\Vert ^{4})\,\exp {(2\boldsymbol{\beta }^T \boldsymbol{Z})}\Big \} \right] <\infty . $$

### Theorem 1

Under Assumptions 1–3, then for every $y_0 \in [a_F, b_F]$,

$$ \lambda _0(y_0^-)\le \liminf _{n\rightarrow \infty }\hat{\lambda }_n(y_0)\le \limsup _{n\rightarrow \infty }\hat{\lambda }_n(y_0)\le \lambda _0(y_0^+) \quad \text {with probability one.} $$

Theorem 1 establishes the pointwise consistency of the proposed estimator at a fixed point $y_0$ in the interior of the support, implying that if $y_0$ is a continuity point of $\lambda _0$, then $\hat{\lambda }_n(y_0)\rightarrow {\lambda }_0(y_0)$ almost surely.

The following theorem establishes the asymptotic distribution of the proposed estimator at a fixed point. To simplify the notation, we define

24 $$\begin{aligned} \Phi (\boldsymbol{\beta }, y) = \int \textbf{1}\{t \le y \le u\} \cdot \exp {(\boldsymbol{\beta }^T \boldsymbol{z})} \, dP(t, u, \delta , \boldsymbol{z}), \end{aligned}$$

for $\boldsymbol{\beta } \in \mathbb {R}^p$, $y \in \mathbb {R}$, where *P* is the underlying probability measure corresponding to the distribution of $(T,Y, \Delta , \boldsymbol{Z})$. In addition, the argmin function refers to the supremum of times at which the minimum is attained.

### Theorem 2

Under Assumptions 1–3, suppose that the baseline hazard $\lambda _0$ is non-decreasing on [0,∞) and continuously differentiable in a neighborhood of $y_0$, with $\lambda _0(y_0) \ne 0$ and $\lambda _0'(y_0) > 0$. Moreover, assume that the sub-distribution function of the uncensored observations $F^{uc}(y):= \mathbb {P}(X \le y|T\le X\le C)$ and $y \mapsto \Phi (\boldsymbol{\beta }_0, y)$ are continuously differentiable in a neighborhood of $y_0$. Then,

25 $$\begin{aligned} n^{1/3} \left( \frac{\Phi (\boldsymbol{\beta }_0, y_0)}{4 \lambda _0(y_0) \lambda _0'(y_0)} \right) ^{1/3} \left( \hat{\lambda }_n(y_0) - \lambda _0(y_0) \right) \xrightarrow {d} \arg \min _{t \in \mathbb {R}} \left\{ \mathbb {W}(t) + t^2 \right\} , \end{aligned}$$

where $\mathbb {W}$ is standard two-sided Brownian motion starting at zero.

The proposed estimator exhibits non-standard asymptotic behavior, with its limiting distribution following the Chernoff distribution and a convergence rate of $n^{1/3}$. This behavior is characteristic of isotonic estimators, where the monotonicity constraint leads to slower convergence rates and non-Gaussian limiting distributions.

## Simulations

### Study Design

We conducted a simulation study to compare our proposed estimator (19) with existing approaches for estimating the survival probability at the true median survival time. Specifically, we consider: (i) the univariate truncated [11] estimator, computed using only subjects with Z=z, without assuming monotone hazards; (ii) the univariate [22] estimator, applied to the subset of subjects with Z=z under the monotone hazard assumption; (iii) the multivariate [3] estimator modified for left truncation, which leverages the full sample through covariates under the [5] PH model but does not assume monotone hazards; and (iv) the multivariate [15] estimator, which incorporates covariates and assumes monotone hazards, but is restricted to right-censored data. Our proposed estimator extends the approach of Lopuhaä and Nane to handle left truncation, reducing to the Tsai estimator in the absence of covariates. Table 1 summarizes the key characteristics of these estimators.

We evaluated the performance of estimators using three metrics: average bias, defined as the difference between the average estimated survival probability at the true median survival time and the true value (0.5); empirical standard error (ESE), defined as the standard deviation of the estimates across simulation replications; and mean squared error (MSE), defined as the average squared difference between the estimated survival probability at the true median survival time and 0.5 across simulation replications. All simulations and analyses were performed using R statistical software (version 4.3.3) and Python (version 3.10). The corresponding program code is available at https://github.com/angelafafa/monohaz_cov.

**Table 1:** Classification of estimators by covariate adjustment, left truncation adjustment, and monotonicity assumption.

	Covariate	Allow left	Monotonicity
**Estimator**	adjustment	truncation	assumption
Truncated KM [23]	No	Yes	No
Tsai [22]	No	Yes	Yes
Breslow [3]	Yes	Yes	No
Lopuhaä [15]	Yes	No	Yes
Proposed	Yes	Yes	Yes

### Data Generating Processes

We adopt the notation from Sect. 2.1. The simulated dataset is $\{T_i, Y_i, \Delta _i, Z_i\},\ i=1,\dots ,n$, where Y=min(X,C) denotes the observed follow-up time with censoring indicator $\Delta =\textbf{1}(X\le C)$, $Z \in \{0,1\}$ indicates gender (0 = male, 1 = female), with the initial sample consisting of $n_0$ males and $n_1$ females, where $n_0 = n_1 = n/2$. For each subject, the event time is $X \sim \textrm{Weibull}(a,\theta _Z)$ and the censoring time is $C \sim \textrm{Weibull}(a,\theta _Z)$, with scale parameters $\theta _0 = 1000$ for males and $\theta _1 = 2000$ for females. Using identical distributions for *X* and *C* within each gender induces approximate 50% censoring. The truncation time is generated as $T \sim \textrm{Weibull}(a,b)$. The shape parameter *a* is shared across the truncation, event, and censoring distributions, while the truncation scale *b* is chosen to yield comparable effective sample sizes across different shape values, since subjects with X<T are excluded by left truncation. With a common shape *a*, the PH assumption holds and the hazard ratio (females vs males) is $(1/2)^a$. The true regression coefficient in the Cox PH model is $\beta = a \log (1/2)$, and the true median survival time for group *Z* is $m_Z = \bigl (-\log 1/2\bigr )^{1/a}\,\theta _Z$. While a=1 corresponds to a constant hazard, a>1 indicates an increasing hazard. We consider four scenarios with shape parameter values a=1,2,4,6, and corresponding truncation scale parameters b=50,250,500,600. For each scenario, we simulate 1000 datasets at two initial sample sizes: n=1000 to assess asymptotic properties and n=100 to highlight the advantage of our proposed estimator over Tsai’s.

To further assess the robustness of these methods, we considered an extreme early-truncation setting with $T\sim \text {Weibull} (4,3000)$ to estimate the baseline survival probability. Simulations were repeated 1000 times with a fixed observed sample size of n=1000, focusing on males, whose smaller scale parameter leads to a higher likelihood of early events. To capture high variability at early times, we repeatedly generated large latent pools of 100000 observations until rare early outliers (T<200) appeared. After removing subjects with X<T, we retained all eligible early outliers and randomly sampled from the remaining eligible population to obtain a final sample of size n=1000. Table 2 shows an illustrative slice of the simulated dataset: subjects 1 and 2 enter very early (T=145,182) and are kept as outliers; subjects 3 and 6 are dropped (T>X); and subjects 4 and 5 enter later (T=300,1200).

**Table 2:** An example of a sliced simulation dataset.

ID	*Z*	*T*	*X*	*C*	*Y*	Δ	Keep T≤Y?	Note
1	0	145	520	800	520	1	Yes	Left outlier (T<200)
2	0	182	310	900	310	1	Yes	Left outlier (T<200)
3	0	700	540	1200	540	1	No	Dropped (left truncated)
4	1	300	1500	1300	1300	0	Yes	Kept
5	1	1200	2500	4000	2500	1	Yes	Kept
6	1	400	380	900	380	1	No	Dropped (left truncated)

Notes: Y=min(X,C) and $\Delta =\textbf{1}\{X\le C\}$. Subjects are observed iff T≤Y

### Results

**Fig. 1:**
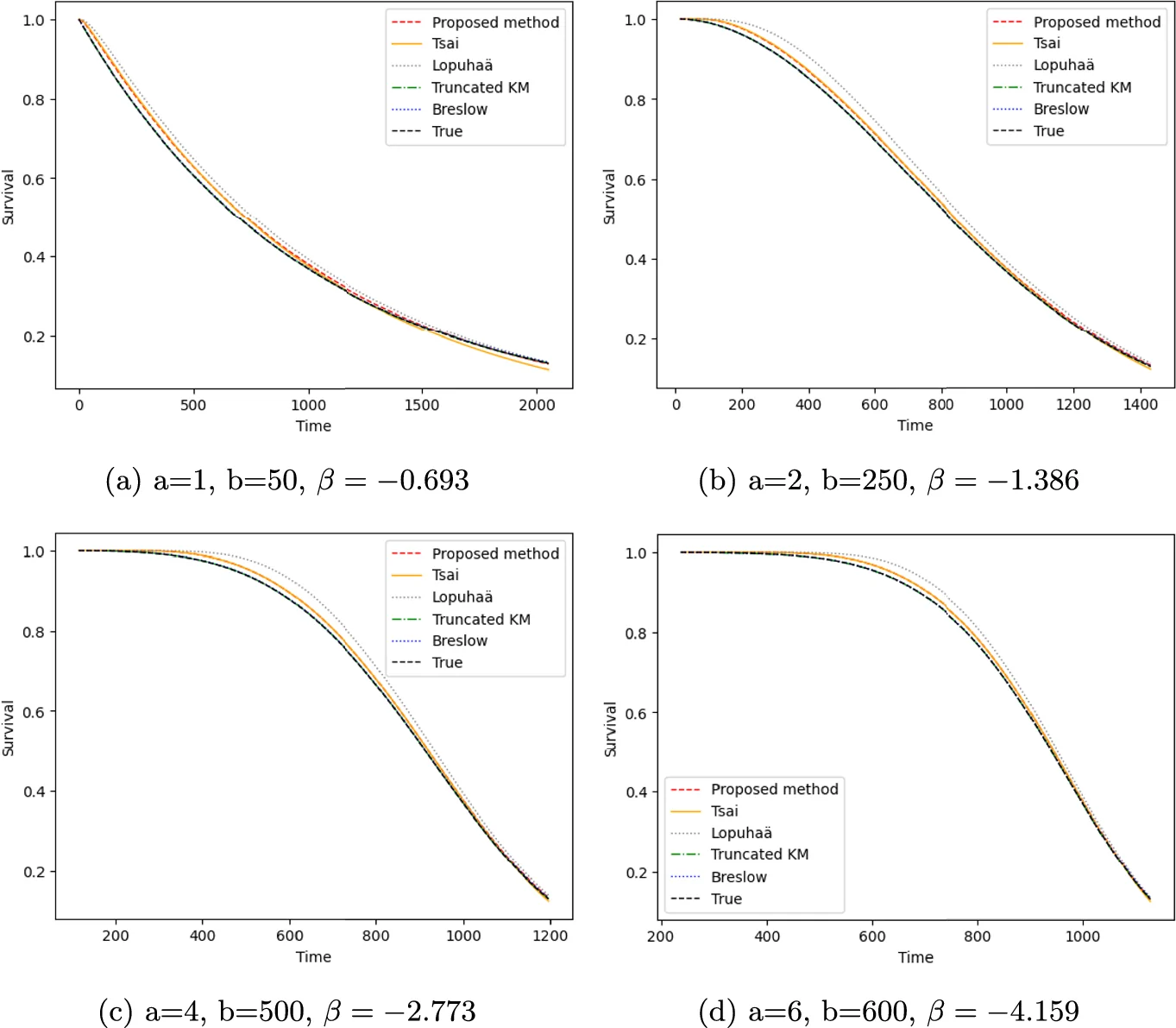
Average predicted survival curves for the male (baseline) subgroup (Z=0), where event times follow a Weibull(*a*, 1000) distribution and truncation times follow a Weibull(a,b) distribution, based on 1000 simulation replicates with initial sample size n=1000. Survival estimates were calculated using the proposed method (red), Tsai estimator (orange), Lopuhaä estimator (grey), truncated KM estimator (green), and Breslow estimator (blue), with the true survival curve shown in black.

**Fig. 2:**
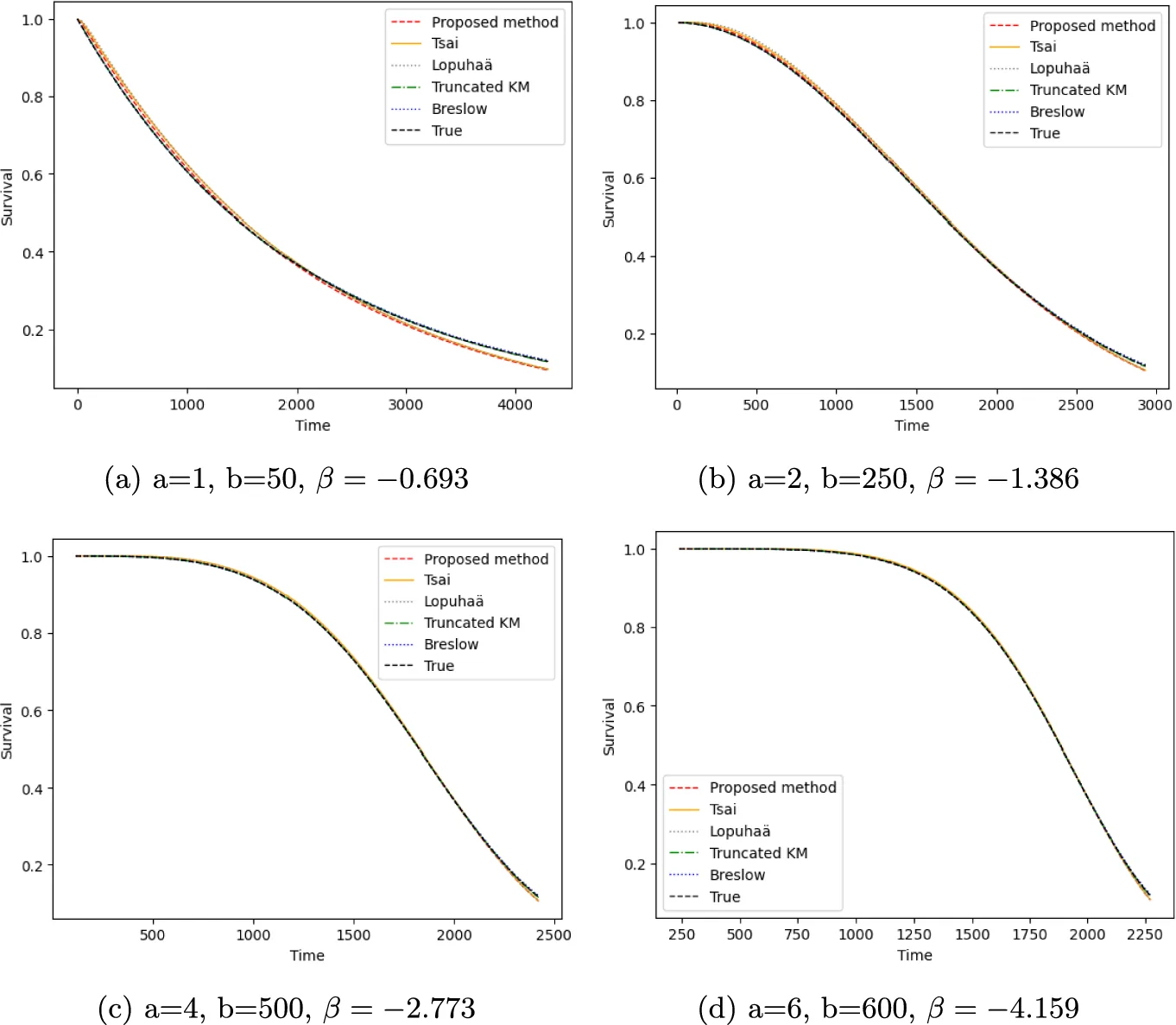
Average predicted survival curves for the female subgroup (Z=1), where event times follow a Weibull(*a*, 2000) distribution and truncation times follow a Weibull(*a*, *b*), based on 1000 simulation replicates with initial sample size n=1000. Survival estimates were calculated using the proposed method (red), Tsai estimator (orange), Lopuhaä estimator (grey), truncated KM estimator (green), and Breslow estimator (blue), with the true survival curve shown in black.

Table 3 summarizes the results from 1000 replications with an initial sample size of n=1000, including the average observed covariate subgroup size $(n_Z)$, the true subgroup-specific median survival time $(m_Z)$, and the average bias, ESE, and MSE of the estimated survival probability at $m_Z$, reported separately for males and females across the five estimators. Although the truncated KM and modified Breslow estimators, both asymptotically unbiased, exhibit smaller biases at the true male median survival time than Tsai and the proposed estimators, the latter two show superior ESE and comparable MSE relative to the non-monotone estimators, and their performance improves as the shape parameter increases, consistent with closer adherence to the monotone hazard assumption. For the female subgroup, the proposed and modified Breslow estimators yield the smallest biases, highlighting the advantage of covariate adjustment under the Cox PH model. In addition to performance at the median survival time, Figures 1 and 2 display the average estimated survival curves for males and females, respectively, across the four scenarios. These curves were obtained by computing estimated survival probabilities at 100 evenly spaced time points for each simulation replicate and then averaging the results across all 1000 replicates with initial sample size n=1000. Figure 1(a) illustrates the boundary case of the monotone assumption at a=1, which introduces a slight increase in bias in both the Tsai and proposed estimators. While the Tsai estimator fails to converge under a constant hazard, the proposed estimator remains more robust, approaching the true value more closely over time by leveraging the full sample through the Cox PH framework. In Figures 1(b)-(d), both the Tsai and proposed estimators slightly underestimate the hazard at early times but converge toward the truth. Lopuhaä estimator, which does not account for left truncation, systematically overestimates baseline survival due to the inclusion of inflated risk sets. In Figure 2, biases are smaller overall for females because their larger scale parameter reduces the impact of sparse early risk sets. Under a constant hazard (a=1), the bias is less pronounced at early times than in males, and the Lopuhaä estimator shows much reduced overestimation. With increasing hazards in Figures 2(b)–(d), all estimators align closely with the true survival curves.

Given that both the Tsai estimator and our proposed estimator are consistent, their performance is nearly equivalent in large samples, as reported in Table 3. However, Tsai’s method requires subsetting to a single stratum, which can lead to inefficiency and instability when subgroup sizes are small. Table 4 reports results from 1000 replications with the initial sample size n=100, presented in the same format as Table 3 except for the reduced sample size. Through covariate adjustment, our proposed estimator achieves improved accuracy and efficiency compared to Tsai’s estimator under small sample size. Notably, the efficiency gap between the Tsai estimator and our proposed estimator narrows as the shape parameter *a* increases. A larger *a* corresponds to a more rapidly increasing hazard function that better satisfies the monotonicity assumption, thereby enhancing the stability of the Tsai estimator. Correspondingly, both the proposed and Tsai estimators generally attain smaller MSE than the non-monotone estimators.

**Fig. 3:**
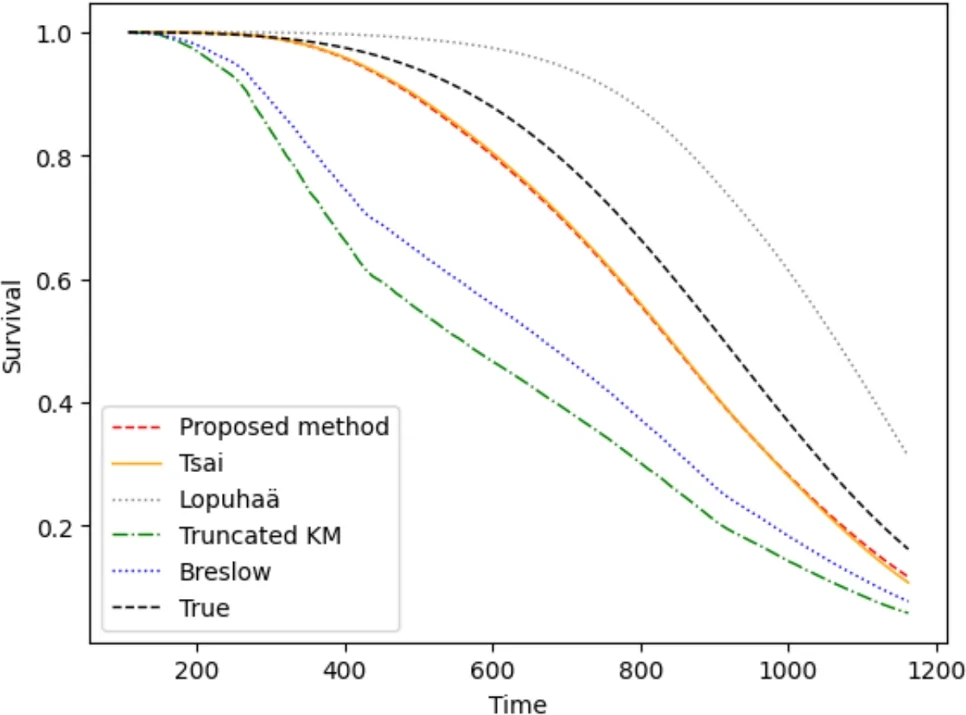
Average estimated survival curves for the male (baseline) subgroup (Z=0), where event times follow a Weibull(4, 1000) distribution and left truncation times were generated from a Weibull(4, 3000) distribution with a few rare early truncation times included as left outliers, based on 1000 simulations with a fixed observed sample size of n=1000. Survival estimates were calculated using the proposed method (red), Tsai estimator (orange), Lopuhaä estimator (grey), truncated KM estimator (green), and Breslow estimator (blue), with the true survival curve shown in black.

Figure 3 highlights the impact of extreme early truncation: small early risk sets make the truncated KM and modified Breslow estimators highly sensitive to early failures. Under the male extreme-truncation setting in Table 3 (a=4, b=3000), both exhibit substantial downward bias and markedly inflated MSE, indicating systematic error. In contrast, the proposed and Tsai estimators have much smaller MSE, with biases remaining comparable to their own ESEs, suggesting that the deviations are mainly due to small-sample fluctuation. Under the same monotonicity assumption, our proposed estimator effectively addresses the small risk-set problem, similar to the Tsai estimator, while additionally leveraging covariate adjustments to improve efficiency. Importantly, the observed bias is primarily induced by artificially sparse early risk sets, rather than reflecting a contradiction of asymptotic properties.

**Table 3:** Average bias, empirical standard error (ESE), and mean squared error (MSE) of the estimated survival probability at the true subgroup-specific median survival time $m_Z$ (for Z=0 and Z=1), based on 1000 simulated datasets with initial sample size n=1000

Scenario	Size	Median	Proposed			Tsai			Lopuhaä			Breslow			Truncated KM		
(*a*, *b*)	$n_Z$	$m_Z$	Bias	ESE	MSE	Bias	ESE	MSE	Bias	ESE	MSE	Bias	ESE	MSE	Bias	ESE	MSE
**Estimated survival probability for males ** $\hat{S}(m_0|Z=0)$																	
(1, 50)	454	693	0.018	0.023	0.001	0.017	0.024	0.001	0.035	0.022	0.002	0.001	0.027	0.001	<0.001	0.032	0.001
(2, 250)	444	833	0.012	0.025	0.001	0.013	0.026	0.001	0.036	0.024	0.002	0.002	0.028	0.001	0.001	0.031	0.001
(4, 500)	444	912	0.011	0.026	0.001	0.011	0.027	0.001	0.035	0.025	0.002	0.001	0.029	0.001	0.001	0.031	0.001
(6, 600)	457	941	0.010	0.026	0.001	0.010	0.027	0.001	0.027	0.026	0.001	0.001	0.030	0.001	<0.001	0.032	0.001
(4, 3000)*	62	912	-0.106	0.077	0.017	-0.105	0.087	0.019	0.244	0.053	0.062	-0.250	0.047	0.065	-0.303	0.125	0.107
**Estimated survival probability for females ** $\hat{S}(m_1|Z=1)$																	
(1, 50)	475	1386	0.004	0.024	0.001	0.012	0.024	0.001	0.013	0.023	0.001	-0.001	0.027	0.001	-0.001	0.028	0.001
(2, 250)	484	1665	<0.001	0.025	0.001	0.006	0.026	0.001	0.006	0.025	0.001	<-0.001	0.027	0.001	-0.001	0.028	0.001
(4, 500)	496	1825	-0.001	0.026	0.001	0.003	0.026	0.001	0.001	0.026	0.001	<-0.001	0.027	0.001	-0.001	0.027	0.001
(6, 600)	499	1881	<-0.001	0.026	0.001	0.002	0.026	0.001	<0.001	0.026	0.001	<-0.001	0.027	0.001	-0.001	0.027	0.001

In this extreme truncation scenario, datasets were resampled to include rare early outliers (T<200) within a fixed observed sample size of n=1000

**Table 4:** Average bias, empirical standard error (ESE), and mean squared error (MSE) of the estimated survival probability at the true subgroup-specific median survival time $m_Z$ (for Z=0 and Z=1), based on 1000 simulated datasets with initial sample size n=100

Scenario	Size	Median	Proposed			Tsai			Lopuhaä			Breslow			Truncated KM		
(*a*, *b*)	$n_Z$	$m_Z$	Bias	ESE	MSE	Bias	ESE	MSE	Bias	ESE	MSE	Bias	ESE	MSE	Bias	ESE	MSE
**Estimated survival probability for males** $\hat{S}(m_0|Z=0)$																	
(1, 50)	45	693	0.043	0.080	0.008	0.039	0.088	0.009	0.053	0.079	0.009	0.002	0.091	0.008	-0.001	0.103	0.011
(2, 250)	44	833	0.034	0.084	0.008	0.031	0.090	0.009	0.050	0.082	0.009	0.005	0.094	0.009	<-0.001	0.103	0.011
(4, 500)	44	912	0.031	0.088	0.009	0.028	0.091	0.009	0.048	0.086	0.010	0.007	0.101	0.010	-0.001	0.106	0.011
(6, 600)	45	941	0.028	0.088	0.008	0.027	0.089	0.009	0.040	0.086	0.009	0.006	0.105	0.011	-0.001	0.106	0.011
**Estimated survival probability for females** $\hat{S}(m_1|Z=1)$																	
(1, 50)	47	1386	0.011	0.078	0.006	0.036	0.082	0.008	0.017	0.077	0.006	0.007	0.086	0.007	0.004	0.093	0.009
(2, 250)	48	1665	0.002	0.079	0.006	0.025	0.083	0.007	0.006	0.079	0.006	0.010	0.088	0.008	0.004	0.092	0.008
(4, 500)	49	1825	0.002	0.081	0.007	0.019	0.083	0.007	0.003	0.081	0.007	0.011	0.092	0.009	0.004	0.093	0.009
(6, 600)	49	1881	-0.003	0.079	0.006	0.010	0.081	0.007	-0.003	0.079	0.006	0.006	0.087	0.008	-0.001	0.089	0.008

## Application

**Fig. 4:**
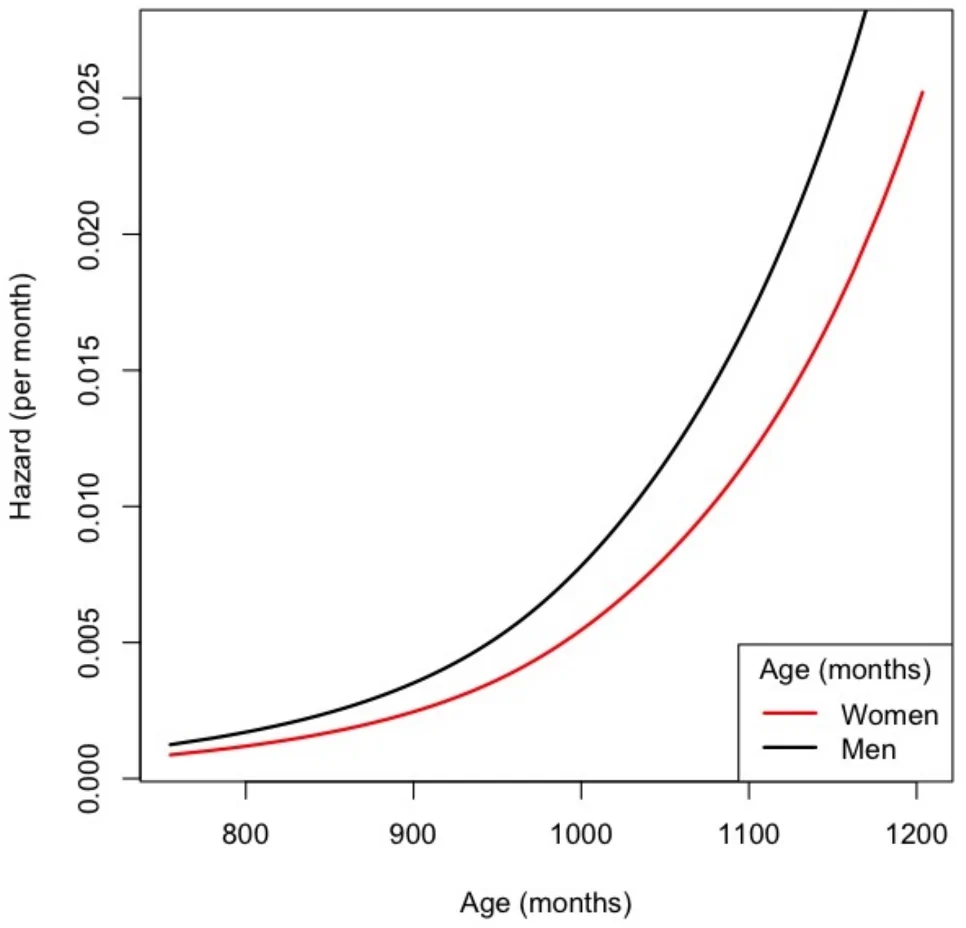
Smoothed hazard of Channing House data assuming proportionality of the hazard in men (black) and women (red).

In this section, we illustrate the application of the estimators discussed above by estimating the survival probabilities for men in the Channing House retirement center in California [9]. Data were collected on 462 elderly residents from the opening of the center in January 1964 through July 1, 1975. During this period, 97 men and 365 women entered the center, with their ages (in months) at entry as well as at exit or death recorded. Left truncation arises because residents are only observed after moving into the facility, meaning individuals who died prior to entry are excluded. Right censoring occurs for residents who either left the center before July 1, 1975, or were still alive on that date. Truncation times are recorded as ages at entry, while censoring times correspond to ages at either withdrawal or the end of the study. After excluding one male subject whose age at entry and exit is identical, 96 male subjects remain for analysis. Among 96 men, 46 died while 50 were right-censored. Figure 4 illustrates that the assumption of an increasing hazard is reasonable, based on a smoothed nonparametric hazard estimate obtained using the bshazard package, which applies B-splines within a generalized linear mixed model framework [20]. Furthermore, the human hazard rate typically follows a U-shaped pattern: high at birth, low during early life, and rising sharply with age. Since most residents enter the study after age 60, the data primarily capture the late-life increasing hazard, consistent with the Gompertz law of mortality [6].

**Fig. 5:**
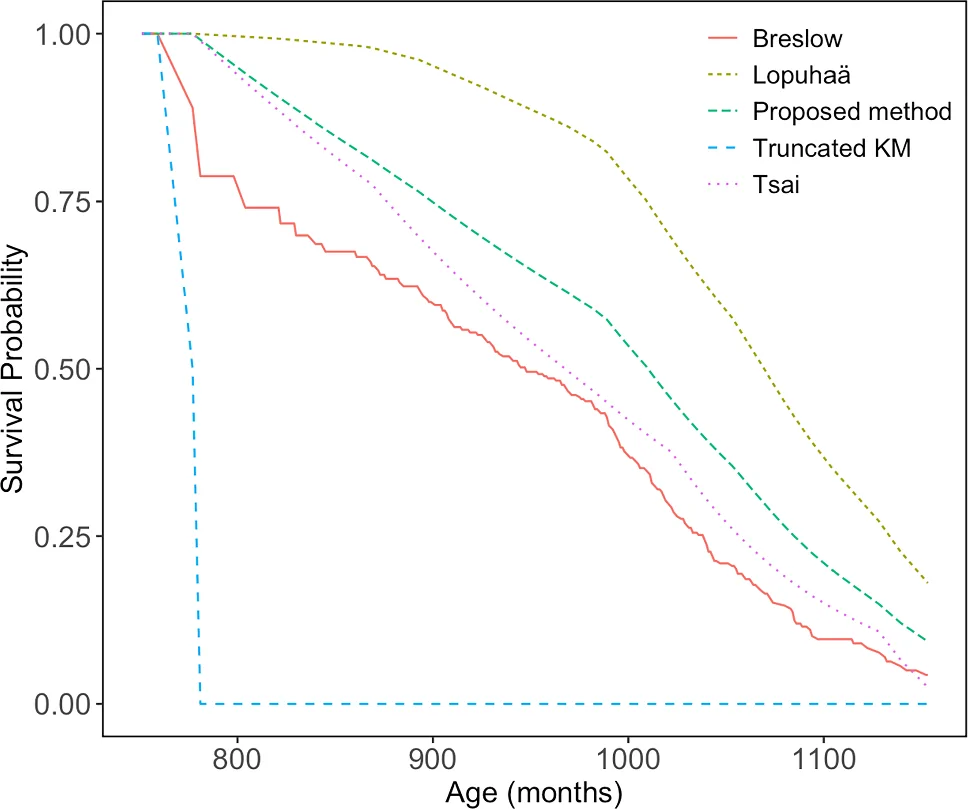
Estimates of survival probabilities for males in Channing House data on estimation methods: our proposed method (cyan), Lopuhaä’s estimator (olive), Tsai’s estimator (purple), Truncated KM estimator (blue), and Breslow’s estimator (red).

As the earliest observed failure occurs at 777 months, the underlying survival function *S* is not identifiable over the interval (0, 777). Similar to Figure 3, Figure 5 also exhibits a sharp initial drop in the survival estimates produced by the truncated [11] and modified [3] estimators. Each of the first two drops, at times 777 and 781, is based on information from only two individuals, suggesting that both non-monotone estimators can be unstable when early-time risk sets are small. In contrast, the monotone estimators of [15, 22], and our proposed method produce continuous survival curves, as integrating continuous hazard functions induces continuous cumulative hazards, exponentiating their negatives therefore forms continuous survival curve estimates. The Lopuhaä estimator, which ignores delayed entry, consistently yields higher survival estimates. The proposed estimator aligns closely with the Tsai estimator but maintains a slightly higher profile, likely due to the inclusion of gender as a single binary covariate, which captures the extended survival times observed in the female demographic.

The Channing House data example is used here to compare Tsai’s univariate estimator for the male cohort with our covariate-adjusted PH baseline estimator for the same cohort. Although our estimator is not strictly necessary in this simple case, its favorable performance-producing results nearly equivalent to those of Tsai’s estimator-is reassuring. Combined with the simulation results presented earlier, this suggests that our method remains effective in more complex PH models involving multiple continuous predictors, where the Tsai estimator is not applicable.

## Discussion

Without monotonicity constraints, discrete hazard estimators can encounter substantial challenges when applied to left-truncated data, regardless of the extent of right censoring. Both the extreme early-truncation setting and the Channing House analysis demonstrate that the truncated [11] and modified [3] estimators severely underestimate the survival function in the presence of small early risk sets. Although both non-monotone estimators are asymptotically unbiased, their sensitivity to small at-risk denominators can lead to dramatic declines in the estimated survival curves. To overcome the instability of discrete hazard estimation under left truncation, we proposed a covariate-adjusted monotone MLE within the [5] PH framework for left-truncated and right-censored data. Unlike non-monotone discrete hazard estimators that rely solely on risk-set counts, our monotone continuous hazard estimator also incorporates total time at risk, yielding more stable early-time estimates. Under the monotone hazard assumption, the proposed method estimates continuous hazard functions (equal to conditional densities) and thus yields continuous survival curves that are smoother than conventional stair-step curves. It also addresses key limitations of existing approaches: the [15] estimator is biased in the presence of left truncation, while the [22] estimator does not adjust for covariates, which would be impractical in multivariable settings. In contrast, our method accommodates both covariates and left truncation by integrating a monotone baseline hazard with a Breslow-type covariate adjustment framework, while modifying the risk sets to account for delayed entry. To facilitate comparisons with univariate estimators, we focus on datasets with two groups, leaving the evaluation of our method in high-dimensional covariate settings to future work. While our method improves estimation efficiency, the monotonicity assumption may not always hold in practice. Therefore, we recommend carefully assessing this assumption before applying the proposed estimator, which is particularly well suited for studies with delayed entry and consequently small early-time risk sets, provided the hazard can be reasonably assumed to be nondecreasing.

## Appendix A Strong consistency

Recall the at-risk and counting processes

$$ R_i(t)=\textbf{1}\{T_i\le t\le Y_i\}, \qquad N_i(t)=\textbf{1}\{Y_i\le t,\ \Delta _i=1\}, $$

and define, for k=0,1,2,

$$ S_n^{(k)}(\boldsymbol{\beta },t) =\frac{1}{n}\sum _{j=1}^n R_j(t)e^{\boldsymbol{\beta }^T \boldsymbol{Z}_j}\boldsymbol{Z}_j^{\otimes k}, \qquad S^{(k)}(\boldsymbol{\beta },t) =\mathbb {E}\!\left[ R(t)e^{\boldsymbol{\beta }^T \boldsymbol{Z}}\boldsymbol{Z}^{\otimes k}\right] . $$

We also write

$${ S_R(y;\boldsymbol{\beta }):=S^{(0)}(\boldsymbol{\beta },y)=\mathbb {E}[R(y)e^{\boldsymbol{\beta }^T \boldsymbol{Z}}], \qquad \alpha _0:=\mathbb {E}[\Delta ], \qquad F_u(y):=\frac{\mathbb {E}[\Delta \textbf{1}\{Y\le y\}]}{\alpha _0}.}$$

Its empirical version is

$${ \hat{S}_R(y;\boldsymbol{\hat{\beta }}_n)=\frac{1}{n}\sum _{i=1}^n R_i(y)\exp (\boldsymbol{\hat{\beta }}_n^T \boldsymbol{z}_i),\qquad \hat{\alpha }_0=\frac{1}{n}\sum _{i=1}^n \delta _i,\qquad \hat{F}_u(y):=\frac{\sum _{i=1}^n \delta _i \textbf{1}\{y_i\le y\}}{\sum _{i=1}^n \delta _i}.} $$

For $$y\in [a_F,b_F]$$, define

$$ H(y;\boldsymbol{\beta }_0)=\int _{a_F}^y \frac{S_R(u;\boldsymbol{\beta }_0)}{\alpha _0}\,du, \qquad \hat{H}_n(y;\hat{\boldsymbol{\beta }}_n)=\int _{a_F}^y \frac{S_n^{(0)}(\hat{\boldsymbol{\beta }}_n,u)}{\hat{\alpha }_0}\,du, $$

and for t∈[0,1],

$$ H(t;\boldsymbol{\beta }_0):=H(F_u^{-1}(t);\boldsymbol{\beta }_0), \qquad H_n(t):=\hat{H}_n(\hat{F}_u^{-1}(t);\hat{\boldsymbol{\beta }}_n). $$

### Lemma 1

Under Assumptions 1– 3, for each k=0,1,2 and each η>0,

$$ \sup _{t\in [a_F,b_F]}\sup _{\Vert \boldsymbol{\beta }-\boldsymbol{\beta }_0\Vert \le \eta } \bigl \Vert S_n^{(k)}(\boldsymbol{\beta },t)-S^{(k)}(\boldsymbol{\beta },t)\bigr \Vert \rightarrow 0 \qquad \text {a.s.} $$

Moreover, for every $M<b_F$, there exists η>0 such that

$$ \inf _{t\in [a_F,M]}\inf _{\Vert \boldsymbol{\beta }-\boldsymbol{\beta }_0\Vert \le \eta } S^{(0)}(\boldsymbol{\beta },t)>0. $$

Hence, almost surely, for all sufficiently large n,

$$ \inf _{t\in [a_F,M]}\inf _{\Vert \boldsymbol{\beta }-\boldsymbol{\beta }_0\Vert \le \eta } S_n^{(0)}(\boldsymbol{\beta },t)>0. $$

### Proof

For fixed k, consider

$$ \mathcal F_k= \Bigl \{(T,Y,\boldsymbol{Z})\mapsto \textbf{1}\{T\le t\le Y\} e^{\boldsymbol{\beta }^T\boldsymbol{Z}}\boldsymbol{Z}^{\otimes k} :\ t\in [a_F,b_F],\ \Vert \boldsymbol{\beta }-\boldsymbol{\beta }_0\Vert \le \eta \Bigr \}. $$

Since

$$ \textbf{1}\{T\le t\le Y\}=\textbf{1}\{T\le t\}\textbf{1}\{Y\ge t\}, $$

the indicator part is a VC class. The class

$$ \Bigl \{\boldsymbol{Z}\mapsto e^{\boldsymbol{\beta }^T\boldsymbol{Z}}\boldsymbol{Z}^{\otimes k} :\ \Vert \boldsymbol{\beta }-\boldsymbol{\beta }_0\Vert \le \eta \Bigr \} $$

is finite-dimensional parametric and hence VC-type (Euclidean). Therefore $\mathcal F_k$ is VC-type. Moreover, by Cauchy–Schwarz and Assumption 3,

$$ E\!\left[ \sup _{\Vert \boldsymbol{\beta }-\boldsymbol{\beta }_0\Vert \le \eta } \Vert \boldsymbol{Z}\Vert ^k e^{\boldsymbol{\beta }^T\boldsymbol{Z}}\right] \le \left( E\!\left[ \sup _{\Vert \boldsymbol{\beta }-\boldsymbol{\beta }_0\Vert \le \eta } \Vert \boldsymbol{Z}\Vert ^{2k} e^{2\boldsymbol{\beta }^T\boldsymbol{Z}}\right] \right) ^{1/2} <\infty , $$

since 2k≤4. Hence $\mathcal F_k$ has an integrable envelope and is Glivenko–Cantelli, yielding the stated uniform convergence.

For each $y\in [a_F,M]$, Assumption 2 implies

$$ \Pr (T\le y\le Y)>0. $$

Since $e^{\boldsymbol{\beta }_0^T \boldsymbol{Z}}>0$, it follows that

$$ S^{(0)}(\boldsymbol{\beta }_0,y) = E\!\left[ \textbf{1}\{T\le y\le Y\}e^{\boldsymbol{\beta }_0^T \boldsymbol{Z}}\right] >0. $$

Because $y\mapsto S^{(0)}(\boldsymbol{\beta }_0,y)$ is continuous on the compact interval $[a_F,M]$,

$$ \inf _{y\in [a_F,M]} S^{(0)}(\boldsymbol{\beta }_0,y)>0. $$

By continuity of $(\boldsymbol{\beta },y)\mapsto S^{(0)}(\boldsymbol{\beta },y)$, we may choose η>0 small enough such that

$$ S^{(0)}(\boldsymbol{\beta },y)>0 \qquad \text {for all } y\in [a_F,M],\ \Vert \boldsymbol{\beta }-\boldsymbol{\beta }_0\Vert \le \eta . $$

Since $\{\boldsymbol{\beta }:\Vert \boldsymbol{\beta }-\boldsymbol{\beta }_0\Vert \le \eta \}\times [a_F,M]$ is compact and $S^{(0)}$ is continuous, we obtain

$$ \inf _{y\in [a_F,M]}\inf _{\Vert \boldsymbol{\beta }-\boldsymbol{\beta }_0\Vert \le \eta }S^{(0)}(\boldsymbol{\beta },y)>0. $$

The final display for $S_n^{(0)}$ follows from the uniform convergence already proved. □

### Lemma 2

Under Assumptions 1– 3, the Cox partial-likelihood estimator with delayed entry satisfies

$$ \hat{\boldsymbol{\beta }}_n\rightarrow \boldsymbol{\beta }_0 \qquad \text {a.s.} $$

Consequently, for every $M<b_F$,

$$ \liminf _{n\rightarrow \infty }\inf _{t\in [a_F,M]} S_n^{(0)}(\hat{\boldsymbol{\beta }}_n,t)>0 \qquad \text {a.s.} $$

### Proof

In the Cox model under left truncation and right censoring, the risk indicator is $R_i(t)=1\{T_i\le t\le Y_i\}$, so the partial likelihood is the standard Cox partial likelihood with delayed-entry risk sets. Strong consistency of $\hat{\boldsymbol{\beta }}_n$ follows from the standard counting-process theory for Cox regression under independent delayed entry.

Fix $M<b_F$, and let η>0 be as in Lemma 1. Since $\hat{\boldsymbol{\beta }}_n\rightarrow \boldsymbol{\beta }_0$ almost surely, we have $\Vert \hat{\boldsymbol{\beta }}_n-\boldsymbol{\beta }_0\Vert \le \eta$ eventually almost surely. The result then follows from Lemma 1. □

### Lemma 3

Assume $F_u$ is continuous and strictly increasing on $[a_F,b_F]$ with range [0,1]. Then

$$ \sup _{t\in [0,1]}|H_n(t)-H(t;\boldsymbol{\beta }_0)|\rightarrow 0 \qquad \text {a.s.} $$

### Proof

By the strong law of large numbers,

$$ \hat{\alpha }_0\rightarrow \alpha _0 \qquad \text {a.s.} $$

Moreover, $\alpha _0>0$. By Lemma 2, $\hat{\boldsymbol{\beta }}_n\rightarrow \boldsymbol{\beta }_0$ almost surely, so eventually $\hat{\boldsymbol{\beta }}_n$ lies in the neighborhood of $\boldsymbol{\beta }_0$ considered in Lemma 1. Hence

$$ \sup _{u\in [a_F,b_F]} |S_n^{(0)}(\hat{\boldsymbol{\beta }}_n,u)-S^{(0)}(\hat{\boldsymbol{\beta }}_n,u)|\rightarrow 0 \qquad \text {a.s.} $$

Since $(\boldsymbol{\beta },u)\mapsto S^{(0)}(\boldsymbol{\beta },u)$ is continuous and $\hat{\boldsymbol{\beta }}_n\rightarrow \boldsymbol{\beta }_0$,

$$ \sup _{u\in [a_F,b_F]} |S^{(0)}(\hat{\boldsymbol{\beta }}_n,u)-S^{(0)}(\boldsymbol{\beta }_0,u)|\rightarrow 0 \qquad \text {a.s.} $$

Therefore,

$$ \sup _{u\in [a_F,b_F]} |S_n^{(0)}(\hat{\boldsymbol{\beta }}_n,u)-S_R(u;\boldsymbol{\beta }_0)|\rightarrow 0 \qquad \text {a.s.} $$

Together with $\hat{\alpha }_0\rightarrow \alpha _0>0$, this yields

$$ \sup _{u\in [a_F,b_F]} \left| \frac{S_n^{(0)}(\hat{\boldsymbol{\beta }}_n,u)}{\hat{\alpha }_0} - \frac{S_R(u;\boldsymbol{\beta }_0)}{\alpha _0} \right| \rightarrow 0 \qquad \text {a.s.} $$

Hence

$$ \sup _{y\in [a_F,b_F]} |\hat{H}_n(y;\hat{\boldsymbol{\beta }}_n)-H(y;\boldsymbol{\beta }_0)| \rightarrow 0 \qquad \text {a.s.} $$

Moreover, $\hat{F}_u\rightarrow F_u$ uniformly almost surely by the Glivenko–Cantelli theorem, and since $F_u$ is continuous and strictly increasing, the generalized inverses satisfy

$$ \sup _{t\in [0,1]}|\hat{F}_u^{-1}(t)-F_u^{-1}(t)|\rightarrow 0 \qquad \text {a.s.} $$

Because $H(\cdot ;\boldsymbol{\beta }_0)$ is continuous on $[a_F,b_F]$, it follows that

$$ \sup _{t\in [0,1]} |H(\hat{F}_u^{-1}(t);\boldsymbol{\beta }_0)-H(F_u^{-1}(t);\boldsymbol{\beta }_0)| \rightarrow 0 \qquad \text {a.s.} $$

Therefore, for every t∈[0,1],

$${ |H_n(t)-H(t;\boldsymbol{\beta }_0)| \le \sup _{y\in [a_F,b_F]}|\hat{H}_n(y;\hat{\boldsymbol{\beta }}_n)-H(y;\boldsymbol{\beta }_0)| + |H(\hat{F}_u^{-1}(t);\boldsymbol{\beta }_0)-H(F_u^{-1}(t);\boldsymbol{\beta }_0)|.} $$

Taking the supremum over t∈[0,1] and using the previous two displays proves the claim. □

### Lemma 4

Assume the Cox PH model holds with delayed entry, and $F_u$ is absolutely continuous with density $f_u$. For every $y\in [a_F,b_F]$ such that $\lambda _0$ is continuous at y and $f_u(y)>0$,

$$ \left[ \frac{d}{dt}H(t;\boldsymbol{\beta }_0)\right] _{t=F_u(y)}=\frac{1}{\lambda _0(y)}. $$

### Proof

Under the Cox model, the compensator of N(y) is

$$ R(y)e^{\boldsymbol{\beta }_0^T \boldsymbol{Z}}\lambda _0(y)\,dy. $$

Taking expectations gives

$$ \mathbb {E}[dN(y)] = S_R(y;\boldsymbol{\beta }_0)\lambda _0(y)\,dy. $$

On the other hand, by definition of $F_u$,

$$ \mathbb {E}[dN(y)] = \alpha _0 f_u(y)\,dy. $$

Thus, for Lebesgue-a.e. y,

$$ \alpha _0 f_u(y)=S_R(y;\boldsymbol{\beta }_0)\lambda _0(y). $$

Now

$$ H(t;\boldsymbol{\beta }_0)=\int _{a_F}^{F_u^{-1}(t)} \frac{S_R(u;\boldsymbol{\beta }_0)}{\alpha _0}\,du, $$

so by the chain rule,

$$ \left[ \frac{d}{dt}H(t;\boldsymbol{\beta }_0)\right] _{t=F_u(y)} = \frac{S_R(y;\boldsymbol{\beta }_0)}{\alpha _0 f_u(y)} = \frac{1}{\lambda _0(y)}. $$

□

### Proof of Theorem 1

Since $$\hat{S}_R(\cdot ;\boldsymbol{\hat{\beta }}_n)$$ is piecewise constant on $$[v_j,v_{j+1})$$, for s=1,…,k,

$$\begin{aligned} \hat{H}_n(v_s;\boldsymbol{\hat{\beta }}_n) = \sum _{j:\,v_j<v_s} \frac{\hat{S}_R(v_j;\boldsymbol{\hat{\beta }}_n)}{\hat{\alpha }_0}\,(v_{j+1}-v_j) = \frac{1}{\sum _{i=1}^n \delta _i}\\ \sum _{j:\,v_j<v_s} \Bigg (\sum _{i\in R(v_j+)} \exp (\boldsymbol{\hat{\beta }}_n^T \boldsymbol{z}_i)\Bigg )(v_{j+1}-v_j). \end{aligned}$$

Therefore, the max–min representation in (19) can be rewritten as

$$ \tilde{\lambda }_j = \max _{0\le r\le j}\; \min _{\begin{array}{c} j\le s\le k-1\\ \hat{F}_u(v_s)>\hat{F}_u(v_r) \end{array}} \frac{\hat{F}_u(v_s)-\hat{F}_u(v_r)}{\hat{H}_n(v_s;\boldsymbol{\hat{\beta }}_n)-\hat{H}_n(v_r;\boldsymbol{\hat{\beta }}_n)}, \qquad j=1,\ldots ,k-1. $$

By the max–min characterization above, $\hat{\lambda }_n$ is the reciprocal of the slope of the GCM of the empirical TTT curve $H_n$, evaluated at the transformed design points $t_s=\hat{F}_u(v_s)$. Since $H_n\rightarrow H(\cdot ;\beta _0)$ uniformly almost surely by Lemma 3, and since $H(\cdot ;\beta _0)$ is concave with left and right derivatives corresponding to $1/\lambda _0$ by Lemma 4, the standard consistency results for slopes of greatest concave minorants imply that, for every $y_0\in [a_F,b_F]$,

$$ \lambda _0(y_0^-)\le \liminf _{n\rightarrow \infty }\hat{\lambda }_n(y_0)\le \limsup _{n\rightarrow \infty }\hat{\lambda }_n(y_0)\le \lambda _0(y_0^+) \quad \text {a.s.} $$

[2, 4]

Hence, at every continuity point $y_0$ of $\lambda _0$,

$$ \hat{\lambda }_n(y_0)\rightarrow \lambda _0(y_0) \qquad \text {a.s.} $$

□

## Appendix B Asymptotic properties

To derive the limit distribution of $\hat{\lambda }_n$, we follow the inverse-process approach of [15]. Define

$$ \hat{U}_n^\lambda (a) = \arg \min _{y\in [v_1,v_k]} \{V_n(y)-a\hat{W}_n(y)\}, \qquad a>0, $$

where

$$ V_n(y)=\int \delta \,\textbf{1}\{t\le u<y\}\,dP_n(t,u,\delta ,\boldsymbol{z}), $$

and let $\hat{\boldsymbol{\beta }}_n$ denote the maximum partial likelihood estimator. Then

$$ \hat{W}_n(y) = W_n(\hat{\boldsymbol{\beta }}_n,y)-W_n(\hat{\boldsymbol{\beta }}_n,v_1), \qquad y\ge v_1 $$

where

$$ W_n(\boldsymbol{\beta },y) = \int \left( e^{\boldsymbol{\beta }^\top \boldsymbol{z}} \int _0^y \textbf{1}\{t\le s\le u\}\,ds \right) dP_n(t,u,\delta ,\boldsymbol{z}). $$

Then the switching relation

$$ \hat{U}_n^\lambda (a)\ge y \iff \hat{\lambda }_n(y)\le a $$

holds almost surely for a>0. Hence, for $y_0\in (a_F,b_F)$,

$$ n^{1/3}\bigl (\hat{\lambda }_n(y_0)-\lambda _0(y_0)\bigr )>a \iff n^{1/3}\Bigl ( \hat{U}_n^\lambda (\lambda _0(y_0)+n^{-1/3}a)-y_0 \Bigr )<0. $$

Define

$$ I_n(y_0)=\bigl [-n^{1/3}(y_0-v_1),\,n^{1/3}(v_k-y_0)\bigr ], $$

and

$$ \Phi (\boldsymbol{\beta },y) = \int \textbf{1}\{t\le y\le u\}e^{\boldsymbol{\beta }^\top \boldsymbol{z}}\,dP(t,u,\delta ,\boldsymbol{z}). $$

Further, define the localized process

$$ \hat{\mathbb Z}_n^\lambda (y) = \frac{n^{2/3}}{\Phi (\boldsymbol{\beta }_0,y_0)} \Bigl ( V_n(y_0+n^{-1/3}y)-V_n(y_0) - \lambda _0(y_0)\bigl [\hat{W}_n(y_0+n^{-1/3}y)-\hat{W}_n(y_0)\bigr ] \Bigr ), $$

for $y\in I_n(y_0)$. After the change of variables $u=y_0+n^{-1/3}y$, subtraction of the *y*-independent constant $V_n(y_0)-\{\lambda _0(y_0)+n^{-1/3}a\}\hat{W}_n(y_0)$, and multiplication by the positive factor $n^{2/3}/\Phi (\boldsymbol{\beta }_0,y_0)$, the argmin criterion becomes

$$\begin{aligned}  &   n^{1/3} \Bigl ( \hat{U}_n^\lambda (\lambda _0(y_0)+n^{-1/3}a)-y_0 \Bigr ) = \arg \min _{y\in I_n(y_0)}\\  &   \left\{ \hat{\mathbb Z}_n^\lambda (y) - \frac{n^{1/3}a}{\Phi (\boldsymbol{\beta }_0,y_0)} \bigl [\hat{W}_n(y_0+n^{-1/3}y)-\hat{W}_n(y_0)\bigr ] \right\} . \end{aligned}$$

### Lemma 5

Under Assumptions 1–3, for every K>0,

$${ \sup _{|y|\le K} n^{1/3} \Bigl | [\hat{W}_n(y_0+n^{-1/3}y)-\hat{W}_n(y_0)] - [W_n(\boldsymbol{\beta }_0,y_0+n^{-1/3}y)-W_n(\boldsymbol{\beta }_0,y_0)] \Bigr | =o_p(1).} $$

### Proof

Write

$$ \Delta _n(y,\boldsymbol{\beta }) = W_n(\boldsymbol{\beta },y_0+n^{-1/3}y)-W_n(\boldsymbol{\beta },y_0), \qquad |y|\le K. $$

Then

$${ [\hat{W}_n(y_0+n^{-1/3}y)-\hat{W}_n(y_0)] - [W_n(\boldsymbol{\beta }_0,y_0+n^{-1/3}y)-W_n(\boldsymbol{\beta }_0,y_0)] = \Delta _n(y,\hat{\boldsymbol{\beta }}_n)-\Delta _n(y,\boldsymbol{\beta }_0).} $$

By Lemma 2, $\hat{\boldsymbol{\beta }}_n\rightarrow \boldsymbol{\beta }_0$ almost surely. Moreover, the map $\boldsymbol{\beta }\mapsto W_n(\boldsymbol{\beta },\cdot )$ is continuously differentiable in a neighborhood of $\boldsymbol{\beta }_0$. Hence, by the mean-value theorem, for each |y|≤K there exists a random point $\tilde{\boldsymbol{\beta }}_{n,y}$ on the line segment joining $\hat{\boldsymbol{\beta }}_n$ and $\boldsymbol{\beta }_0$ such that

$$ \Delta _n(y,\hat{\boldsymbol{\beta }}_n)-\Delta _n(y,\boldsymbol{\beta }_0) = (\hat{\boldsymbol{\beta }}_n-\boldsymbol{\beta }_0)^\top \Bigl [ \dot{W}_n(\tilde{\boldsymbol{\beta }}_{n,y},y_0+n^{-1/3}y) - \dot{W}_n(\tilde{\boldsymbol{\beta }}_{n,y},y_0) \Bigr ], $$

where $$\dot{W}_n(\boldsymbol{\beta },y)=\partial W_n(\boldsymbol{\beta },y)/\partial \boldsymbol{\beta }$$.

Now

$$ \dot{W}_n(\boldsymbol{\beta },y) = \int \left( \boldsymbol{z}\,e^{\boldsymbol{\beta }^\top \boldsymbol{z}} \int _0^y \textbf{1}\{t\le s\le u\}\,ds \right) dP_n(t,u,\delta ,\boldsymbol{z}), $$

so that

$$ \frac{\partial }{\partial y}\dot{W}_n(\boldsymbol{\beta },y) = \int \boldsymbol{z}\,e^{\boldsymbol{\beta }^\top \boldsymbol{z}}\textbf{1}\{t\le y\le u\} \,dP_n(t,u,\delta ,\boldsymbol{z}). $$

By Assumptions 1–3 and the same argument as in the corresponding plug-in reduction step of [15], it follows that, on every compact neighborhood of $y_0$, the derivative $\partial \dot{W}_n(\boldsymbol{\beta },y)/\partial y$ is uniformly $O_p(1)$ for $\boldsymbol{\beta }$ in a neighborhood of $\boldsymbol{\beta }_0$. It follows that

$$ \sup _{|y|\le K} \Bigl \Vert \dot{W}_n(\tilde{\boldsymbol{\beta }}_{n,y},y_0+n^{-1/3}y) - \dot{W}_n(\tilde{\boldsymbol{\beta }}_{n,y},y_0) \Bigr \Vert = O_p(n^{-1/3}). $$

Consequently,

$${ \sup _{|y|\le K} n^{1/3} \Bigl | \Delta _n(y,\hat{\boldsymbol{\beta }}_n)-\Delta _n(y,\boldsymbol{\beta }_0) \Bigr | \le n^{1/3}\Vert \hat{\boldsymbol{\beta }}_n-\boldsymbol{\beta }_0\Vert \sup _{|y|\le K} \Bigl \Vert \dot{W}_n(\tilde{\boldsymbol{\beta }}_{n,y},y_0+n^{-1/3}y) - \dot{W}_n(\tilde{\boldsymbol{\beta }}_{n,y},y_0) \Bigr \Vert = o_p(1),} $$

since $$\hat{\boldsymbol{\beta }}_n-\boldsymbol{\beta }_0=o_p(1)$$. □

### Proposition 3

Suppose Assumptions 1–3 hold. Assume that $\lambda _0$ is nondecreasing and continuously differentiable in a neighborhood of $y_0\in (a_F,b_F)$, with $\lambda _0(y_0)\ne 0$ and $\lambda _0'(y_0)>0$, and that $y\mapsto \Phi (\boldsymbol{\beta }_0,y)$ is continuously differentiable in a neighborhood of $y_0$. Then

$$ \hat{\mathbb Z}_n^\lambda \Rightarrow \mathbb Z \qquad \text {in } B_{\textrm{loc}}(\mathbb R), $$

where

$$ \mathbb Z(y) = \mathbb W\!\left( \frac{\lambda _0(y_0)}{\Phi (\boldsymbol{\beta }_0,y_0)}\,y\right) +\frac{1}{2}\lambda _0'(y_0)y^2, $$

and W is standard two-sided Brownian motion.

### Proof

By Lemma 5, $\hat{W}_n$ may be replaced by $W_n(\boldsymbol{\beta }_0,\cdot )$ at the $n^{-1/3}$ local scale. Thus it suffices to study

$${ \tilde{\mathbb Z}_n^\lambda (y) = \frac{n^{2/3}}{\Phi (\boldsymbol{\beta }_0,y_0)} \Bigl ( V_n(y_0+n^{-1/3}y)-V_n(y_0) - \lambda _0(y_0)\bigl [ W_n(\boldsymbol{\beta }_0,y_0+n^{-1/3}y)-W_n(\boldsymbol{\beta }_0,y_0) \bigr ] \Bigr ).} $$

The remainder of the proof follows the same local-process argument as in [15], with the right-censoring risk functional replaced by the left-truncation functional

$$ \Phi (\boldsymbol{\beta }_0,y) = \int \textbf{1}\{t\le y\le u\}e^{\boldsymbol{\beta }_0^\top \boldsymbol{z}}\, dP(t,u,\delta ,\boldsymbol{z}). $$

Indeed,

$$ \frac{d}{dy}E\{V_n(y)\}=\lambda _0(y)\Phi (\boldsymbol{\beta }_0,y), \qquad \frac{d}{dy}E\{W_n(\boldsymbol{\beta }_0,y)\}=\Phi (\boldsymbol{\beta }_0,y), $$

so

$$ \frac{d}{dy} \Bigl ( E\{V_n(y)\}-\lambda _0(y_0)E\{W_n(\boldsymbol{\beta }_0,y)\} \Bigr ) = (\lambda _0(y)-\lambda _0(y_0))\Phi (\boldsymbol{\beta }_0,y). $$

Since $\lambda _0$ and $\Phi (\boldsymbol{\beta }_0,\cdot )$ are continuously differentiable at $y_0$, a Taylor expansion yields, uniformly on compact sets,

$$ E\{\tilde{\mathbb Z}_n^\lambda (y)\}\rightarrow \frac{1}{2}\lambda _0'(y_0)y^2. $$

For the centered part, the same shrinking-increment empirical-process argument as in Section 5 of [15] applies. Delayed entry changes only the local risk indicator, replacing the original risk-set term by $\textbf{1}\{t\le s\le u\}$, and hence only the local variance coefficient changes, from the original risk functional to $\Phi (\boldsymbol{\beta }_0,y_0)$. Therefore the centered process converges in $B_{\textrm{loc}}(\mathbb R)$ to

$$ \mathbb W\!\left( \frac{\lambda _0(y_0)}{\Phi (\boldsymbol{\beta }_0,y_0)}\,y\right) , $$

where W is standard two-sided Brownian motion.

Thus Proposition 3 is the left-truncation analogue of the corresponding local-process weak convergence result in [15]; the proof is unchanged except that the risk indicator is replaced by $\textbf{1}\{t\le s\le u\}$, which changes only the local variance coefficient to $\Phi (\boldsymbol{\beta }_0,y_0)$. Combining the deterministic and stochastic parts yields

$$ \hat{\mathbb Z}_n^\lambda \Rightarrow \mathbb Z \qquad \text {in } B_{\textrm{loc}}(\mathbb R), $$

with

$$ \mathbb Z(y) = \mathbb W\!\left( \frac{\lambda _0(y_0)}{\Phi (\boldsymbol{\beta }_0,y_0)}\,y\right) +\frac{1}{2}\lambda _0'(y_0)y^2. $$

□

### Lemma 6

Under the assumptions of Proposition 3, for every $M_1>0$,

$$ \sup _{|a|\le M_1} \left| n^{1/3} \Bigl ( \hat{U}_n^\lambda (\lambda _0(y_0)+n^{-1/3}a)-y_0 \Bigr ) \right| = O_p(1). $$

### Proof

For $$|a|\le M_1$$, define

$$ \hat{\mathbb Z}_n^\lambda (a,y) = \hat{\mathbb Z}_n^\lambda (y) - \frac{n^{1/3}a}{\Phi (\boldsymbol{\beta }_0,y_0)} \bigl [\hat{W}_n(y_0+n^{-1/3}y)-\hat{W}_n(y_0)\bigr ]. $$

By Proposition 3, Lemma 5, and the same argument as in the proof of the corresponding boundedness lemma in [15], we have

$$ \hat{\mathbb Z}_n^\lambda (a,\cdot ) \Rightarrow y\mapsto \mathbb W\!\left( \frac{\lambda _0(y_0)}{\Phi (\boldsymbol{\beta }_0,y_0)}\,y\right) +\frac{1}{2}\lambda _0'(y_0)y^2-ay $$

in $B_{\textrm{loc}}(\mathbb R)$, uniformly for $|a|\le M_1$.

Since $\lambda _0'(y_0)>0$, the quadratic drift dominates outside compact sets, uniformly in $|a|\le M_1$, and therefore the argmin of $\hat{\mathbb Z}_n^\lambda (a,\cdot )$ is bounded in probability. □

### Proof of Theorem 2

For fixed $a\in \mathbb R$, define

$$ \hat{\mathbb Z}_n^\lambda (a,y) = \hat{\mathbb Z}_n^\lambda (y) - \frac{n^{1/3}a}{\Phi (\boldsymbol{\beta }_0,y_0)} \bigl [\hat{W}_n(y_0+n^{-1/3}y)-\hat{W}_n(y_0)\bigr ], \qquad y\in I_n(y_0), $$

and extend it constantly outside $I_n(y_0)$. By Proposition 3 and Lemma 5,

$$ \hat{\mathbb Z}_n^\lambda (a,\cdot )\Rightarrow \bigl [y\mapsto \mathbb Z(y)-ay\bigr ] \qquad \text {in } B_{\textrm{loc}}(\mathbb R), $$

where

$$ \mathbb Z(y) = \mathbb W\!\left( \frac{\lambda _0(y_0)}{\Phi (\boldsymbol{\beta }_0,y_0)}\,y\right) +\frac{1}{2}\lambda _0'(y_0)y^2. $$

By Lemma 6, the associated inverse process is bounded in probability. Hence, exactly as in the proof of Theorem 2 of [15], Theorem 2.7 of [12] together with Theorem 6.1 of [8] yields

$$ n^{1/3} \Bigl ( \hat{U}_n^\lambda (\lambda _0(y_0)+n^{-1/3}a)-y_0 \Bigr ) \Rightarrow U^\lambda (a) := \arg \min _{y\in \mathbb R}\{\mathbb Z(y)-ay\}. $$

Completing the square and using the stationarity of Brownian increments gives

$$ U^\lambda (a)\overset{d}{=}U^\lambda (0)+\frac{a}{\lambda _0'(y_0)}. $$

Moreover, by the switching relation,

$$ \Pr \!\left( n^{1/3}(\hat{\lambda }_n(y_0)-\lambda _0(y_0))>a \right) = \Pr \!\left( n^{1/3} \Bigl ( \hat{U}_n^\lambda (\lambda _0(y_0)+n^{-1/3}a)-y_0 \Bigr )<0 \right) . $$

Therefore,

$$ \Pr \!\left( n^{1/3}(\hat{\lambda }_n(y_0)-\lambda _0(y_0))>a \right) \rightarrow \Pr \!\left( U^\lambda (0)<-\frac{a}{\lambda _0'(y_0)} \right) , $$

and hence

$$ n^{1/3}\bigl (\hat{\lambda }_n(y_0)-\lambda _0(y_0)\bigr ) \Rightarrow -\lambda _0'(y_0)\,U^\lambda (0). $$

Equivalently,

$$ n^{1/3}\bigl (\hat{\lambda }_n(y_0)-\lambda _0(y_0)\bigr ) \Rightarrow -\lambda _0'(y_0)\, \arg \min _{y\in \mathbb R} \left\{ \mathbb W\!\left( \frac{\lambda _0(y_0)}{\Phi (\boldsymbol{\beta }_0,y_0)}\,y\right) +\frac{1}{2}\lambda _0'(y_0)y^2 \right\} . $$

Finally, by Brownian scaling,

$$ n^{1/3}\mathcal A(y_0)\bigl (\hat{\lambda }_n(y_0)-\lambda _0(y_0)\bigr ) \Rightarrow \arg \min _{t\in \mathbb R}\{\mathbb W(t)+t^2\}, $$

where

$$ \mathcal A(y_0) = \left( \frac{\Phi (\boldsymbol{\beta }_0,y_0)}{4\lambda _0(y_0)\lambda _0'(y_0)} \right) ^{1/3}. $$

Thus the limit law is the standard Chernoff distribution. □

## Declarations

### Code availability

The code reproducing the results presented is available in the GitHub repository at https://github.com/angelafafa/monohaz_cov.
